# Factor Analysis-Based Quantitative Endotyping Improves Associations With CRS Cross-Sectional and Longitudinal Outcomes

**DOI:** 10.1002/alr.70027

**Published:** 2025-09-26

**Authors:** Brooke N. Gleason, Zhidi Luo, Aditi Agarwal, Siyuan Dong, Regan L. Harmon, Junqin Bai, Chun-Kang Liao, Julia Huang, David B. Conley, Kevin C. Welch, Robert C. Kern, Stephanie S. Smith, Anju T. Peters, Whitney S. Stevens, Atsushi Kato, Lutfiyya N. Muhammad, Bruce K. Tan

**Affiliations:** 1 Department of Otolaryngology, Northwestern University Feinberg School of Medicine, Chicago, Illinois, USA; 2 Department of Psychiatry and Behavioral Science, Northwestern University Feinberg School of Medicine, Chicago, Illinois, USA; 3 Department of Preventive Medicine (Biostatistics and Informatics), Northwestern University Feinberg School of Medicine, Chicago, Illinois, USA; 4 Division of Allergy and Immunology, Department of Medicine, Northwestern University Feinberg School of Medicine, Chicago, Illinois, USA

**Keywords:** biomarker, chronic rhinosinusitis, disease severity, endoscopic sinus surgery, endotype, patient reported outcomes

## Abstract

**Background:**

Chronic rhinosinusitis (CRS) is a heterogeneous inflammatory disease characterized by persistent sinonasal inflammation. There is increasing interest in endotype-based classification, which categorizes CRS based on underlying inflammatory pathways. We applied factor analysis to facilitate continuous endotype assignment to CRS patients and assess cross-sectional and longitudinal CRS outcomes.

**Methods:**

We prospectively enrolled 203 patients (52 controls, 88 CRSsNP, and 63 CRSwNP) undergoing endoscopic nasal or sinus surgery (ESS). Middle meatal mucus biomarkers were analyzed pre-ESS (V0) and 6–12 months post-ESS (V1). Factor analysis was performed to identify latent factors. Factor scores were generated, and statistical analyses were conducted to assess correlations with radiographic (Lund–Mackay [LM]) and patient-reported (SNOT-22, CRS-PRO) outcomes measured at V0, V1, and V2 (1.5–5 years post-ESS).

**Results:**

Four factors were identified: Type 1 (T1), Type 2 (T2), Type 3 (T3), and macrophage-associated (M). CRSwNP patients had higher T2 and M factor scores than CRSsNP and controls. T2, T3, and M factor scores demonstrated stronger or equivalent associations with radiographic and patient-reported outcomes compared to individual biomarkers. Following ESS, median T2 and M factor scores significantly declined, while T1 and T3 remained stable. V0 T1 and T2 were weakly associated with long-term (V2) radiographic and symptom scores. V1 factor scores were more consistently predictive of long-term (V2) outcomes, with T2, T3, and M demonstrating modest correlations with radiographic severity and CRS-PRO.

**Conclusions:**

Factor analysis identifies distinct, quantifiable patterns of inflammation in CRS, offering improved associations with cross-sectional and longitudinal outcomes compared to individual biomarkers.

## 1 | Background

Chronic rhinosinusitis (CRS) is a heterogeneous inflammatory disorder of the sinus and paranasal sinus mucosa for at least 12 weeks marked by overlapping symptoms, including facial pain, nasal obstruction, drainage, and reduced sense of smell [1]. Traditionally, CRS has been categorized based on the presence (CRSwNP) or absence (CRSsNP) of nasal polyps. Diagnosis remains clinical, relying on component symptom duration paired with observable inflammation via endoscopic or imaging findings. However, the expanding repertoire of highly targeted biologic therapies has fueled interest in classifying CRS by the molecular nature of the inflammation or endotype. This spurs further need to understand the relationship of endotype to patient outcomes, especially in regard to treatment-specific outcomes. Endoscopic sinus surgery (ESS) remains the first-line treatment for severe CRS, and understanding how endotype influences outcomes is of continued interest.

CRS endotyping work has used two broad approaches: supervised (concept-based) and unsupervised (data-driven). Supervised endotyping utilizes conceptual biological pathways or biomarkers to categorize CRS, often classifying based on single representative cytokines or histologic indicators and binary comparisons (e.g., T2 vs. non-T2): with conceptual inflammation types Type 1 (T1) characterized by IFN-*γ*, Type 2 (T2) characterized by IL-4, IL-5, and IL-13, and Type 3 (T3) characterized by IL-17 [2–4]. Unsupervised methods leverage machine learning and statistical algorithms to analyze large datasets, including biomarker profiles, to uncover hidden endotype patterns affording classification analyses of multiple groups [5]. These approaches have generally used hierarchical clustering and K-means clustering to group patients based on similarities in inflammatory signatures. Both approaches have consistently linked T2-dominant clusters to CRSwNP, asthma, olfactory dysfunction, and increased radiographic severity [6–8]. To date, endotype classification has prioritized grouping patients by shared inflammatory profiles, but this approach prevents inflammation from being assessed along a continuum of severity, imposes discrete group boundaries, and potentially oversimplifies the biological heterogeneity. Furthermore, given the longitudinal nature of disease, some patient classification techniques limit the ability to track dynamic changes in inflammation over time.

A key challenge is how to define endotypes within a multidimensional biomarker space, as more comprehensive biomarker panels may provide a more thorough assessment of CRS inflammation than those defined by singular representative biomarkers. Biomarkers conceptually linked to an endotype pathway may not be strongly correlated or associated with several endotypes. Among T2 biomarkers, IL-13 and IL-5 demonstrate strong, stable correlations, but are only weakly or uncorrelated with other T2 biomarkers like IL-4 and IgE [9]. Additionally, eosinophilic cationic protein (ECP) correlates moderately with T2 cytokines, but it does not predict clinical response to dupilumab, a T2 inflammation inhibitor, in patients with CRSwNP [9, 10]. These limitations underscore the need for multi-biomarker approaches to capture CRS endotypes more accurately.

Multi-biomarker endotyping poses unique complexities; many prior CRS studies have employed principal component analysis (PCA) to qualitatively identify endotypes [6–8, 11], and these components have sometimes been used quantitatively to inform endotyping using clustering algorithms [8, 12]. Interpretation of a PCA requires understanding of both the strength of biomarker loading as well as its directionality, but directionality (e.g., T2 vs. non-T2) is often flattened by rotational algorithms, resulting in PCs whose biological meaning is abstruse. Hierarchical or other agglomerative clustering is then commonly used to organize patients and also biomarkers, but clustering algorithms are sensitive to outliers, inherently unstable, and in the case of hierarchical clustering, result in different numbers of clusters of patients or endotypes that can be generated depending on the sample [13–15]. Factor analysis is an alternative dimension-reducing unsupervised method that aims to uncover discrete underlying, observed factors that explain relationships between observed variables [16, 17]. Compared to PCA, factor analysis can model latent biological mechanisms and delineate complex interactions between biomarker variables. Factor analysis has been successfully applied in CRS research to identify patterns in patient-reported outcome measures (PROMs), such as the 12-item CRS-PRO [18], and Questionnaire of Olfactory Disorders-Negative Statements (QOD-NS) [19]. We noted its application in a study of plasma brain injury and chronic kidney disease (CKD) biomarkers [20, 21] to quantitatively organize biomarker panels. As CRS is a multifaceted disease with interconnected underlying biological processes, we sought to apply factor analysis to CRS biomarkers to uncover latent factors representing biological processes driving CRS pathogenesis. We then further assessed whether factor analysis-derived factor scores better correlated with CRS disease severity and longitudinal outcomes. Throughout this analysis, we further compared our results to the utility of individual biomarkers

## 2 | Materials and Methods

### 2.1 | Study Population and Study Design

**FIGURE 1 |:**
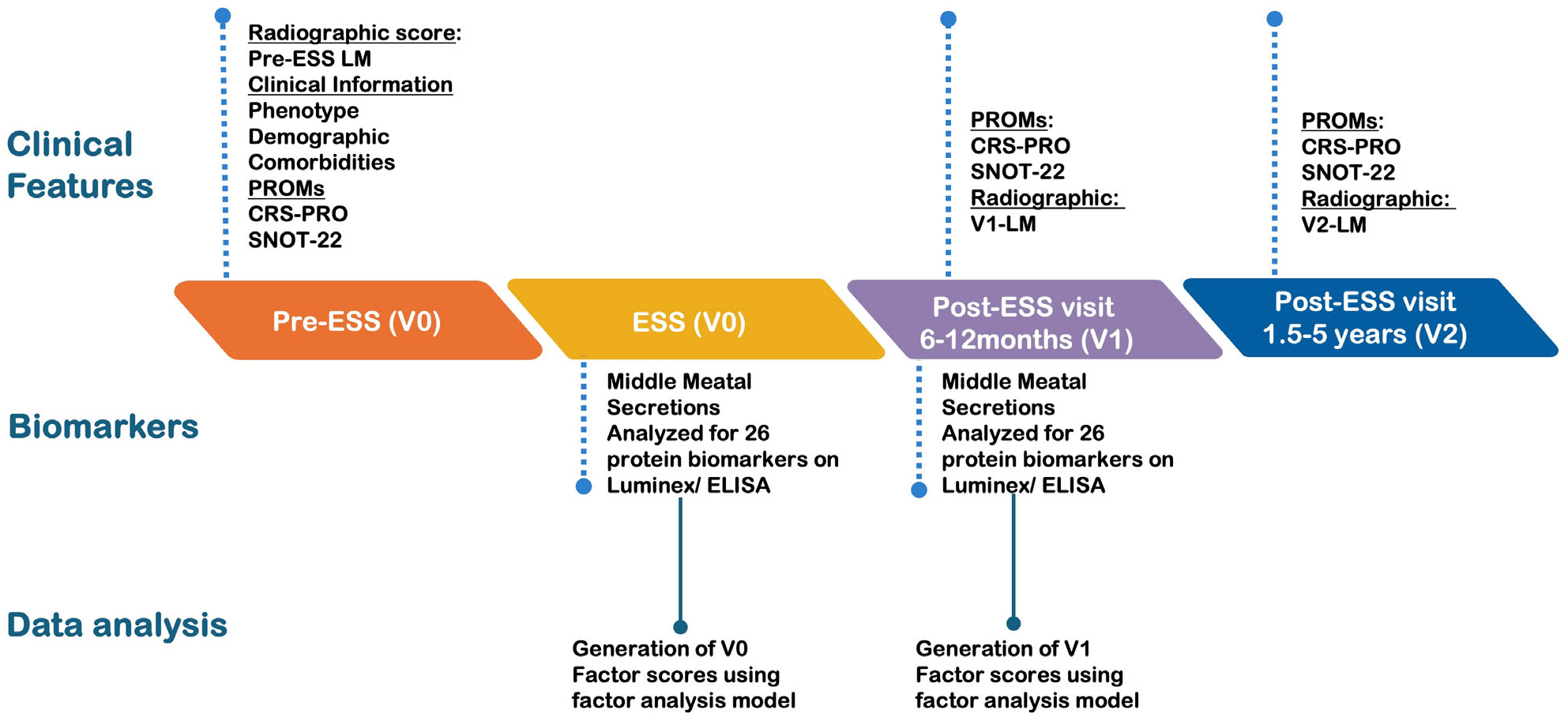
Study design illustrating the timeline for the collection of biomarkers and clinical variables.

Patients with CRSsNP or CRSwNP undergoing ESS at Northwestern Memorial Hospital between 2017 and 2021 were prospectively enrolled. Assessments occurred pre-ESS (V0), 6–12 months after ESS (V1), and 1.5–5 years after ESS (V2) (Figure 1). Participants provided written consent to evaluate nasal secretions and clinical data. Both primary and revision ESS patients were included; exclusions included non-English-speaking patients, pregnancy, an established immunodeficiency, coagulation disorder, or a diagnosis of classic allergic fungal sinusitis. Patients with perioperative corticosteroid use were not excluded from this study. Fifty-two CRS-free control patients provided samples during nasal surgeries for non-CRS indications, but were not followed postoperatively. The Northwestern IRB reviewed this study and approved this protocol (IRB STU00202510).

### 2.2 | Collection of Nasal Secretions

Nasal secretions were collected at time points V0 and V1. Pre-punched 3/8-inch hydroxylated polyvinyl acetate sponges (Medtronic, Independence, OH) were placed in the middle meatus (MM) using endoscopic guidance and left in place for 8–10 min before removal. Samples were stored and processed as previously described [9].

### 2.3 | Collection of Clinical Outcome Measures

Clinical outcomes were assessed at V0, V1, and V2. Previously validated measures included the Lund–Mackay (LM) score for radiographic severity (range: 0–24) [22, 23] and two patient-reported outcome measures (PROMs): the validated 22-item Sinonasal Outcomes Test (SNOT-22), with a score range of 0–110, and the 12-item Chronic Rhinosinusitis Patient-Reported Outcomes Measure (CRS-PRO), which scores between 0 and 48 [18, 24, 25]. In all clinical outcome measures, higher scores indicate greater severity.

### 2.4 | Measurement of Biomarkers

Twenty-six biomarkers were measured. Luminex analysis (MILLIPLEX MAP Human High Sensitivity T-Cell Panel, Merck KGaA, Darmstadt, Germany) was used to quantify levels of IFN*γ*, IL-1*β*, IL-4, IL-5, IL-6, IL-10, IL-13, IL-17A, IL-21, CXCL11, TNF*α*, MIP-1*α*, MIP-1*β*, and MIP-3*α* at a 1:2 dilution. Luminex analysis was utilized to measure levels of periostin, tenascinC, OSM, CXCL9, CXCL13, CCL26, C5a, and TNSF13B/BAFF (Luminex Human Discovery Assay, R&D Systems, Minneapolis, MN) at a 1:10 dilution, as well as IgE (MILLIPLEX MAP Human Immunoglobulin IgE Single Plex, Merck KGaA) at a 1:10 dilution. Levels of tPA were measured by ELISA (Human tPA ELISA Kit, Invitrogen, Waltham, MA) at a 1:10 dilution. HNE levels were measured by ELISA (Human Neutrophil Elastase/ELA2 DuoSet ELISA, R&D Systems, Minneapolis, MN) at a 1:100 dilution. The concentration of ECP was calculated as previously described [9]. Missing values in the biomarker data were analyzed, excluding cases with more than 10% missing data and retaining those with less than 10% missing. Multiple Imputation by Chained Equations (MICE) using the random forest method, which fills in missing values based on patterns in the observed data, was applied to include cases. After imputation, biomarker values were log-transformed to normalize skewed distributions, and standardized by subtracting the mean and dividing by the standard deviation to ensure comparability across biomarkers.

### 2.5 | Factor Analysis Model and Generation of Factor Scores

Exploratory factor analysis (EFA) was performed to uncover underlying structures within the biomarker data. EFA identifies latent factors by analyzing how biomarkers group together based on shared variance. We used varimax rotation, an orthogonal method that simplifies factor loadings by maximizing the variance of squared loadings, making the factors easier to interpret.

The number of factors was determined through parallel analysis scree plots, which compare observed eigenvalues to those from random data to identify meaningful factors. In EFA, biomarkers can load onto multiple factors (cross-loading), and factor loadings reflect the strength of association between each biomarker and a given factor.

Following EFA, we conducted confirmatory factor analysis (CFA) to restrict each biomarker to load onto only one factor, eliminating cross-loadings and providing a more defined factor structure. Biomarkers with EFA loadings greater than 0.4 were included in the CFA model. Factor loadings in CFA are calculated by estimating the strength of the linear relationship between each biomarker and its assigned latent factor. These loadings represent how much a biomarker contributes to the factor. Factor scores were computed for each patient as weighted sums of the standardized biomarker values, where the weights are the corresponding CFA factor loadings, reflecting each individual’s position on the latent factors derived from the biomarker data.

### 2.6 | Statistical Analysis

Statistical analyses were conducted using R version 4.2.2 (Springer-Verlag, New York) and GraphPad Prism software version 10 (La Jolla, CA). Normality was evaluated using the Shapiro–Wilk test. Continuous data are presented as medians and ranges for skewed distributed variables and as means with standard deviations (SDs) for normally distributed variables. Categorical data are reported as frequency counts and percentages. The Pearson chi-square test was applied to assess differences in categorical variables between groups. The Kruskal–Wallis test with Dunn’s post hoc multiple comparisons was used to compare medians across more than two groups at the same time point. A two-tailed *p*-value less than 0.05 was considered statistically significant for the analyses described. Spearman rho correlations were performed to assess relationships between variables. For interpreting correlation strength, values between 0.25 and 0.39 were classified as weak, between 0.40 and 0.69 as moderate, and greater than 0.70 as strong [26–28]. The Wilcoxon signed-rank test for paired samples was utilized to compare repeated measures of factor scores from the same subjects at different time points. The Mann–Whitney *U* test and independent *t*-tests were used to compare medians and means between two groups, respectively.

## 3 | Results

### 3.1 | Study Population

A total of 203 patients were included: 52 CRS-free controls and 151 patients with CRS (88 CRSsNP and 63 CRSwNP) who underwent ESS. The patients with CRS were prospectively enrolled, and their follow-up visits took place at an average of 8.1 (2.3) months and 2.8 (1.1) years after ESS. The average age of the cohort was 46.1 (14.9) years, and there was no significant difference between controls and patients with either CRSsNP and CRSwNP. Detailed comparison of demographic, comorbid, and V0 cross-sectional data is provided in Table 1.

### 3.2 | Factor Analysis

**FIGURE 2 |:**
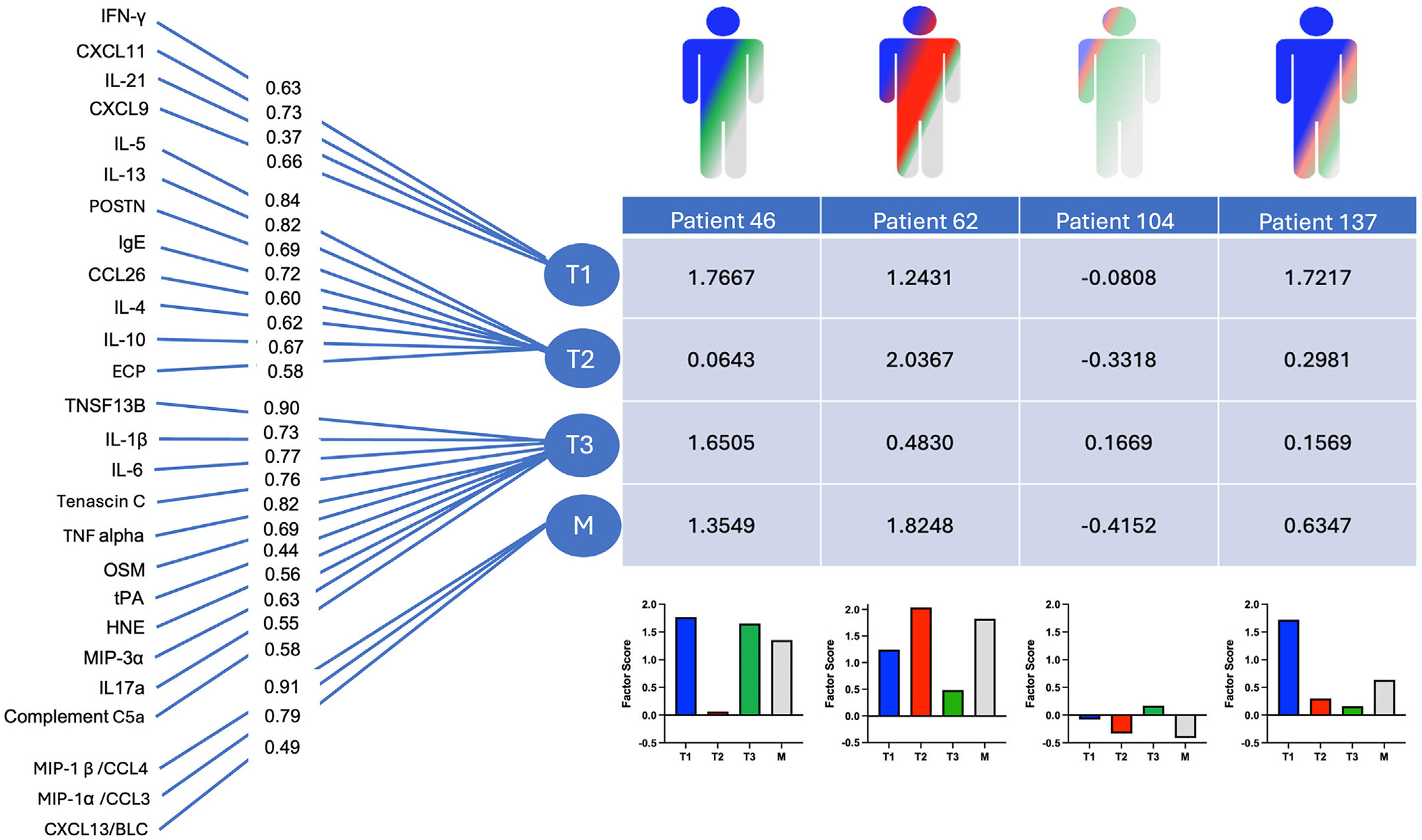
Factor analysis model and four representative factor scores generated. Exploratory factor analysis organized 26 middle meatus secretion biomarkers into four distinct factors identifiable as Type 1 (T1), Type 2 (T2), Type 3 (T3), and macrophage-associated (M). Confirmatory factor analysis restricted biomarkers to load onto one factor and reassigned factor loadings. Factor loadings represent how much a biomarker contributes to the factor and are demonstrated as the numbers overlaying the lines. Factor scores were computed as weighted sums of the standard biomarker values. Four subjects’ factor score profiles are visualized to demonstrate heterogeneity.

EFA was performed on 26 middle meatus secretion biomarkers collected at V0 (Figure 2). Parallel analysis scree plots identified four meaningful factors within the measured biomarkers (Figure S1). IFN-*γ* loaded onto a distinct factor along with IFN-*γ* inducible chemokines like CXCL9 (MIG, monokine induced by interferon gamma) and CXCL11 (ITAC, interferon-gamma inducible T-cell alpha chemoattractant), which we recognize as characteristic of T1 inflammation. Likewise, IL-4, IL-5, and IL-13, and characteristic chemokines like CCL26 (eotaxin-3) loaded onto a single factor recognizable as T2. A third factor loaded canonical T3 biomarkers, including IL17a and the chemokine CCL20 (MIP-3a), but also biomarkers associated with complement activation (C5a), proinflammatory cytokines like IL6, TNF-*α*, and IL1b, and neutrophils (HNE). Notably, CCL3/MIP-1*α*, CCL4/MIP-1*β*, and CXCL13/BLC all loaded onto a separate factor, and we recognize these as chemokines secreted by macrophages (M) [29–31].

### 3.3 | Factor Scores Variation Across CRS Phenotypes

**FIGURE 3 |:**
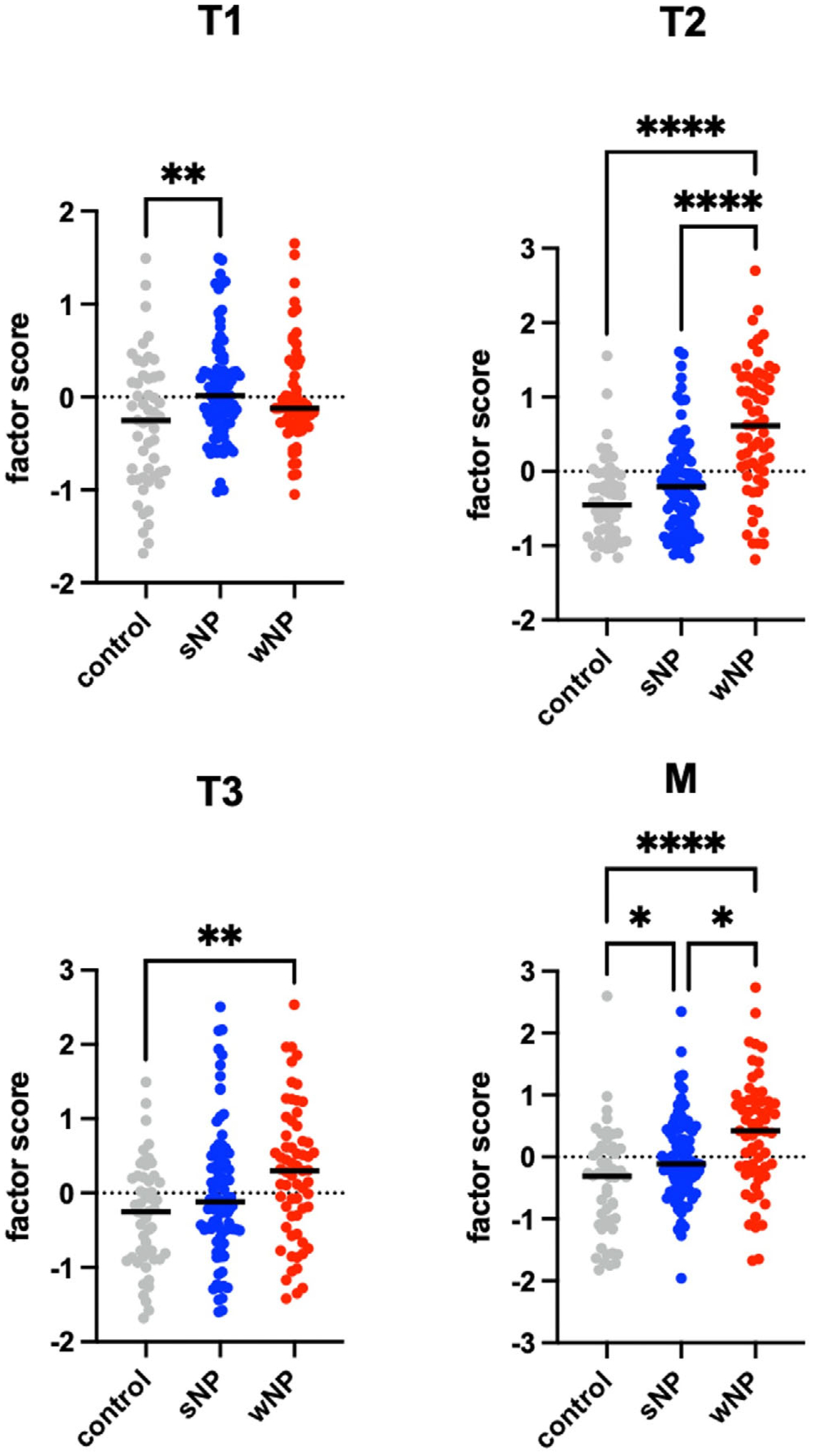
Comparison of V0 factor scores of all identified factors stratified by CRS and CRS phenotype status. Results are shown as medians. *p < 0.05, **p < 0.01, ***p < 0.001, ****p < 0.0001 according to the Kruskal–Wallis test with Dunn multiple comparison. For the entire cohort, *n* = 203; 52 for control, 88 for CRSsNP, and 63 for CRSwNP.

Factor scores for each of the four factors were generated for each participant at V0 and V1 using the product of CFA-generated factor loadings and the biomarkers measured for each patient sample. Factor scores for the four identified factors were stratified by disease type (control, CRSsNP, or CRSwNP) and compared at the V0 timepoint (Figure 3). We found the T1 factor score was the only factor specifically higher in CRSsNP patients, which was significantly greater than the control group (*p* < 0.01). Reassuringly, the CRSwNP patients had significantly greater T2 factor scores than both CRSsNP and control groups (*p* < 0.001). T3 was also significantly greater in CRSwNP patients compared to controls (*p* < 0.01), but there was no difference between CRS phenotypes. M factor scores were significantly different between groups, with CRSwNP showing higher values compared to CRSsNP (*p* = 0.01) and control (*p* < 0.001), and CRSsNP showing higher values compared to control (*p* = 0.03). Detailed factor score summary information is provided in Table 2. There was no significant difference in factor scores between CRSsNP and CRSwNP phenotypes at V1.

### 3.4 | Association of Factor Scores With Cross-Sectional Outcomes and Performance Compared to Individual Biomarkers

**FIGURE 4 |:**
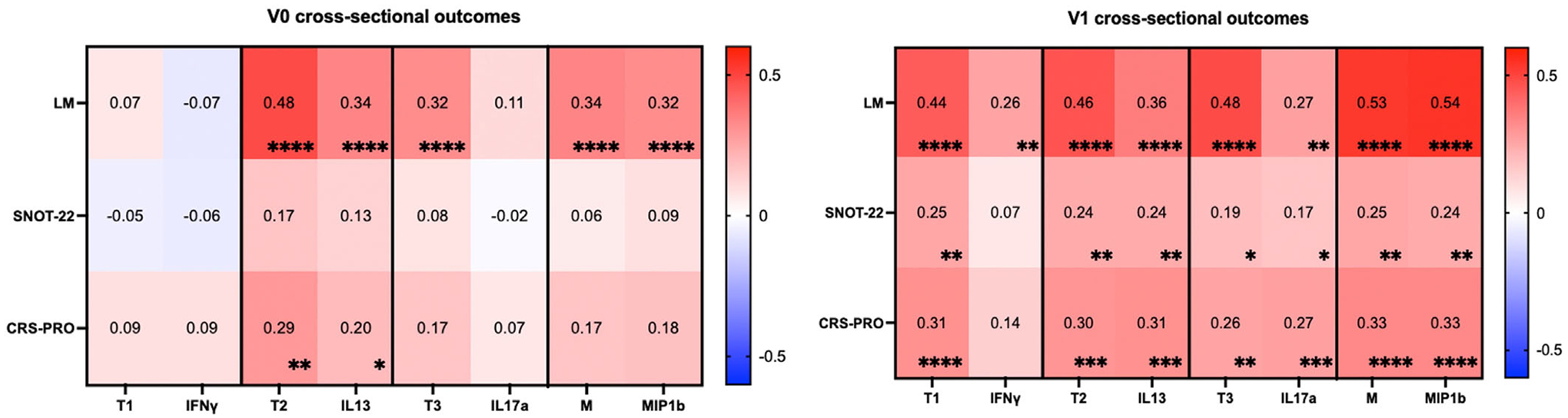
Cross-sectional correlations between factors and individual biomarkers with radiographic and patient-reported measures of disease severity before and after ESS. The intensity of red and blue strength increases as the strength of correlation becomes positive or negative, respectively, as represented in the heatmap. Spearman *R* values are shown in the center of the heatmap, and significance is shown in the bottom right corner. *p < 0.05, **p < 0.01, ***p < 0.001, ****p < 0.0001. V0 LM, *N* = 147; SNOT-22, *N* = 104; CRS-PRO, *N* = 96. For V1, *N* = 148 for all clinical outcome measures.

We next compared endotype factor scores with individual endotype representative biomarkers for their association with cross-sectional clinical outcomes at V0 and V1 (Figure 4). At V0, the T2, T3, and M factor scores were significantly positively associated with LM scores, with stronger relationships noted for the T2 factor score (*r* = 0.48, *p* < 0.001). Notably, the factor scores were consistently more strongly associated with LM than any endotype respective individual biomarker. Likewise, the T2 factor score correlated better with the CRS-PRO (*r* = 0.29, *p* < 0.01) compared to IL-13 alone (*r* = 0.020, *p* = 0.049), and the T3 factor score correlated with LM scores (*r* = 0.32, *p* < 0.001), whereas IL17*α* did not correlate with any clinical outcomes.

At V1, all four-factor scores had even stronger and highly significant cross-sectional associations, especially with radiographic severity, compared to those observed at V0. In contrast to V0 relationships, the CRS-PRO also demonstrated significant positive correlation with all four identified factor scores. With both LM and PROMs, we observed stronger relationships between the factor score and the clinical outcome compared to using individual biomarkers. In particular, T1 demonstrated greater associations with LM (*r* = 0.44, *p* < 0.001), SNOT-22 (*r* = 0.25, *p* < 0.01), and CRS-PRO (*r* = 0.31, *p* < 0.001), compared to IFN-*γ*, which only weakly associated with LM scores (*r* = 0.26, *p* < 0.01). Where significant relationships were observed, the CRS-PRO had a stronger and more significant association with endotype factor scores than the SNOT-22.

### 3.5 | Effect of Sinus Surgery on Factor Scores

**FIGURE 5 |:**
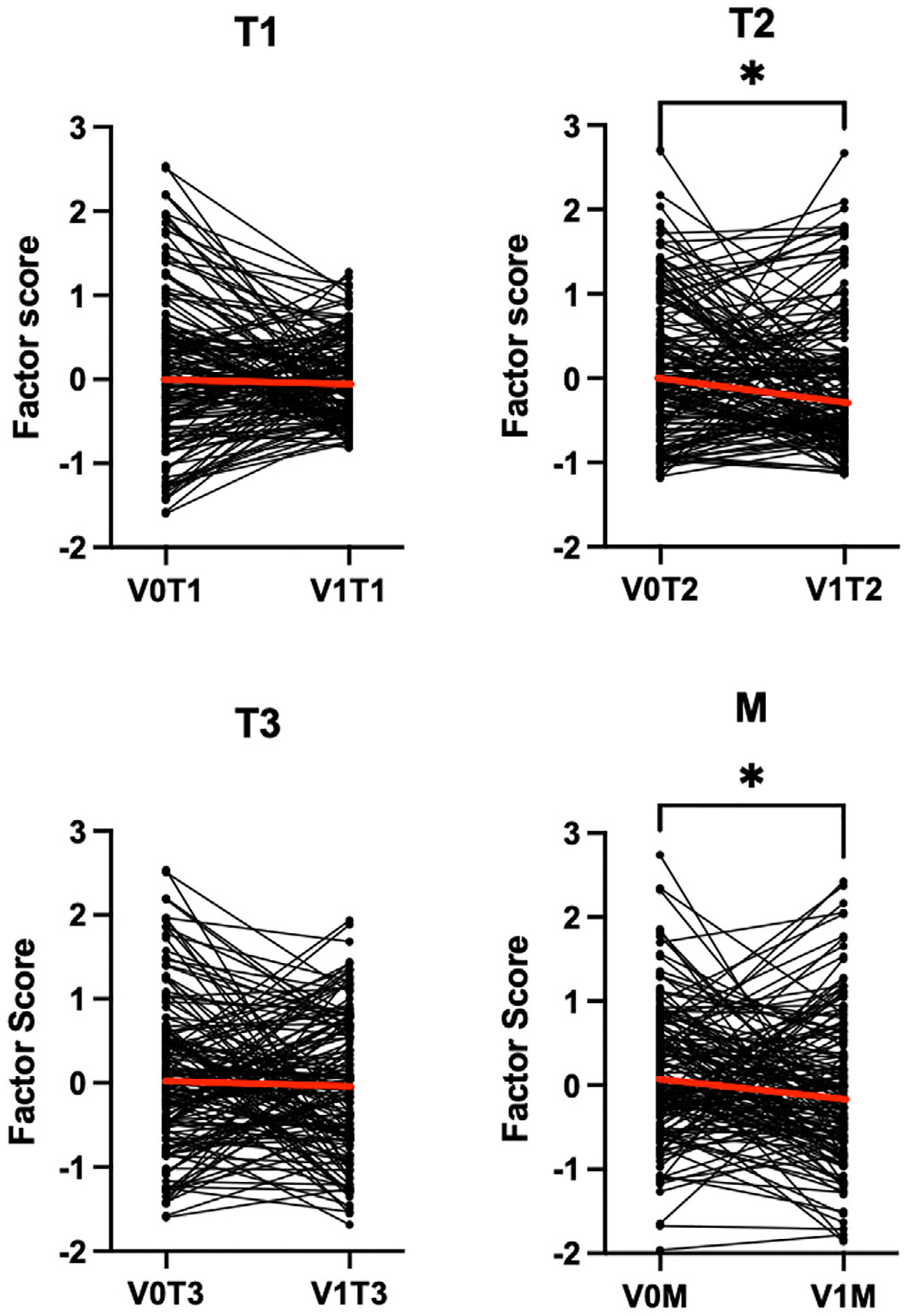
Comparison of factor scores across time points from V0 to V1. T2 and M factor scores decreased significantly following ESS. Lines denote median factor scores. *p < 0.05, **p < 0.01, ***p < 0.001, ****p < 0.0001 by Wilcoxon rank-sum test. *N* = 149.

To understand the effect of sinus surgery on CRS inflammation, we evaluated factor score changes from V0 to V1 (Figure 5). While all factors displayed a decrease in the median level, only T2 (*p* = 0.049) and M (*p* = 0.02) demonstrated a significant decrease in scores between the two time points.

We further evaluated the change in endotype factor scores and the relationship with changes in clinical outcomes between V0 and V1 (Figure S2). This analysis found that changes in LM radiographic severity were consistently correlated with the changes in all four-factor scores, with T2 and M showing stronger relationships. Likewise, CRS-PRO total scores were significantly associated with T2 and M, but were not significantly associated with T1 or T3 scores. There were no significant associations between changes in any factor score and SNOT-22.

### 3.6 | Factor Scores for Predicting Long-Term Outcomes

**FIGURE 6 |:**
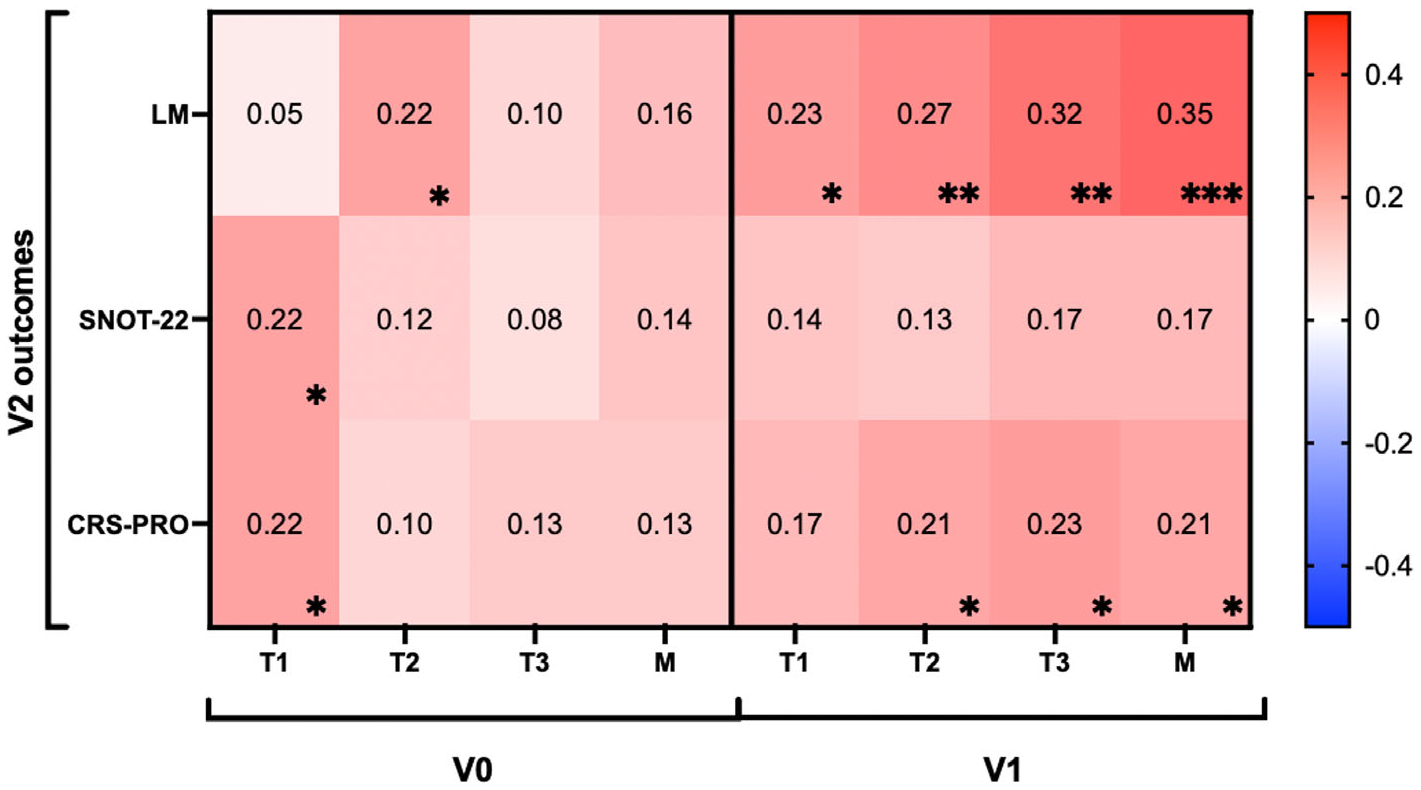
Longitudinal correlations between V0 and V1 factors with V2 radiographic and patient-reported measures of disease severity. The intensity of red and blue strength increases as the strength of correlation becomes positive or negative, respectively, as represented in the heatmap. Spearman *R* values are shown in the center of the heatmap, and significance is shown in the bottom right corner. *p < 0.05, **p < 0.01, ***p < 0.001, ****p < 0.0001. *N* = 97.

We next examined the utility of V0 and V1 factor scores for *predicting* their respective *subsequent* V1 and V2 outcomes. Factor scores at the time of surgery (V0) were generally not associated with V1 outcomes, with only the T1 factor score at V0 weakly associated with V1 (Figure S3) and V2 CRS-PRO (*r* = 0.22, *p* = 0.03) (Figure 6). T2 factor scores at V0 were also associated with LM scores at V2 (*r* = 0.22, *p* = 0.03).

All four V1 factor scores consistently predicted V2 LM severity (*r* = 0.23, *p* = 0.03; *r* = 0.27, *p* < 0.01; *r* = 0.32, *p* < 0.01; *r* = 0.35, *p* < 0.001). Additionally, factor scores for T2, T3, and M were significantly correlated with CRS-PRO (*r* = 0.21, *p* = 0.04; *r* = 0.23, *p* = 0.02; *r* = 0.21, *p* = 0.03). No V1 factor scores predicted SNOT-22 at V2. V0 factor scores associations with V1 outcomes are provided in Figure S3.

## 4 | Discussion

This study investigated whether factor analysis could be applied to dissect the complex interplay of biomarkers in CRS. We applied an unsupervised factor analysis to 26 middle meatus biomarkers and identified four latent factors aligning with T1, T2, T3, and macrophage-related inflammation. These factors allowed the assignment of quantitative, continuous factor scores generated from an individual patient’s nasal secretion biomarker profile. Importantly, factor scores reflected well-established endotype–phenotype associations and demonstrated stronger correlations with cross-sectional clinical outcomes compared to individual biomarkers. They also captured the known effects of ESS on inflammation. This study uniquely uses endotypes as quantitative continuous measures rather than clusters.

Factor analysis identified the conceptual endotypes of CRS, separating T1, T2, and T3 inflammatory responses in agreement with prior studies. Well-established Type 2 cytokines, IL-4, IL-5, and IL-13, organized into the T2 factor alongside periostin, IgE, CCL26, and ECP [2, 32–35]. However, some biomarkers diverged from canonical expectations. A few biomarkers not typically characterized as T3-specific, such as C5a, TNFSF13B (BAFF), and tPA, unexpectedly loaded onto the T3 factor. Likewise, a biomarker for neutrophils, HNE, is loaded onto the T3 factor, despite neutrophils historically being linked to T1 inflammation. In contrast, IL-21, typically associated with T3 [36], is loaded onto the T1 factor. IL-10, an inhibitory cytokine used in many inflammatory pathways loaded onto the T2 factor. Macrophage-related chemokines (MIP-1*α*/*β*, CXCL13) formed a separate factor, suggesting that macrophages influence inflammation independent of traditional T1, T2, and T3 classifications. Together, these findings demonstrate the potential for factor analysis to classify inflammation biomarkers within a CRS-specific context.

Differences in factor scores between controls and CRS phenotypes at V0 highlight distinct phenotype-specific inflammatory profiles. Notably, T1 factor scores are significantly elevated in CRSsNP patients compared to controls, suggesting T1 inflammation may be unique to CRSsNP and contrasts with our previous analysis that found no difference between IFN-*γ* levels among control, CRSsNP, and CRSwNP tissue [37]. However, the previous study assessed only a single biomarker (IFN-*γ*) in tissue, not nasal secretions. The significantly elevated T2 scores in CRSwNP compared to both CRSsNP and controls align well with established literature linking CRSwNP to a dominant Type 2 inflammatory response [6]. We found that T3 was significantly greater in CRSwNP patients compared to controls but not different between CRS phenotypes. This aligns with a similar study, which found that IL-17a was significantly elevated in CRSwNP tissue compared to healthy controls [38]; however, we found no difference in T3 scores between CRS phenotypes, suggesting heterogeneity within CRS. We identified a significant stepwise increase in M factor scores from control to CRSsNP to CRSwNP. A prior study found that CCL3 and CCL4 were highly expressed in nasal fluid samples from eosinophilic CRSwNP patients compared to control patients [39]. Similarly, in a separate study, CXCL13 (BCA-1) was found to be significantly elevated in CRSwNP polyp tissue compared to control and CRSsNP inferior turbinate tissues [40]. Here, we identified a stepwise increase in our M factor scores between CRS phenotypes, potentially reflecting the escalating involvement of key macrophage-associated chemokines, particularly in the more severe phenotypes of the disease.

We assessed associations between factor scores and cross-sectional outcomes, and found that radiographic severity was strongly correlated with inflammation severity, particularly with the T2, T3, and M endotypes across assessed time points. This aligns with our previous finding that Type 2 biomarkers correlated more strongly with LM scores than patient-reported outcomes at all time points, and also demonstrates that T3 and M endotypes may also associate with radiographic severity [9]. Interestingly, at V1, all identified factors had an association with radiographic and PROM severity. Compared to our prior published analysis using individual T2 biomarkers, we found that the factor score frequently outperformed individual representative biomarkers for correlation with radiographic and PROM severity [9, 41]. This study also uniquely identifies significant cross-sectional correlations between non-Type 2 endotypes and PROM total scores, especially after ESS. A prior study found no correlations between any middle meatus mucus biomarkers and total SNOT-22 scores before surgery [42]. Our study similarly found no correlations between individual biomarkers or factor scores with SNOT-22 at V0, but we did identify significant correlations with CRS-PRO. Overall, these findings underscore the benefit of utilizing a multi-biomarker endotype over individual representative biomarkers.

We assessed how ESS impacted factor scores. T2 and M were significantly reduced between V0 and V1, suggesting that specific inflammatory pathways may be more responsive to endoscopic sinus surgery. This is consistent with our prior finding that IL-13, periostin, and CCL26 significantly decreased following ESS [9]. We identified that the novel macrophage-associated factor also significantly decreased following ESS. There are no studies that have measured the change in CCL3, CCL4, or CXCL13 levels following ESS. We also previously reported that reductions in IL-13, periostin, and ECP were associated with decreases in LM scores [9]. In this study, we similarly found that these reductions in T2 and M correlated with improvements in radiographic severity. Additionally, reductions in T2 and M were associated with improvements in CRS-PRO across time points, indicating that the observed reductions may translate to tangible improvements in symptoms and quality of life. Factor scores offer a longitudinal metric, unlike clustering, which lacks numerical tracking. These findings suggest that T2 and M factors may serve as indicators of treatment efficacy or disease progression in CRS.

A major aspiration of endotyping has been to improve prognostication of outcomes following treatment. V0 and V1 factor scores and clinical outcomes at V2 provide insight into how early inflammatory profiles can help predict disease progression and symptom severity in CRS years after ESS. Interestingly, V0 T1 factor scores were associated with SNOT-22 and CRS-PRO at V2, suggesting that the inflammatory profiles may have a lasting impact on both patient-reported symptoms and quality of life over time. The link between elevated baseline T1 inflammation and long-term sinonasal symptoms may stem from a reduced response to common CRS treatments, such as corticosteroids [43], or the continued presence of bacterial biofilms, which may contribute to ongoing symptoms [44]. Similarly, V0 T2 scores were associated with V2 LM severity, indicating that Type 2 inflammation at baseline could be predictive of radiographic disease severity in the future. This pre-ESS T2 prediction of radiographic severity has similar implications to the JESREC study and our prior studies, which found that tissue eosinophilia and T2 inflammation at the time of ESS were significantly associated with recurrence of CRS in the following years [45, 46].

While all V1 factors were significant predictors of radiographic severity at V2, V1 M demonstrated the strongest and most significant prediction. Our finding that the macrophage-associated factor significantly predicts LM severity aligns with a prior study that identified MIP-1*β* as a significant predictor of difficult-to-treat CRS status after 1 year of follow-up [47]. Interestingly, V1 T2, T3, and M demonstrated correlations with CRS-PRO at V2, but not SNOT-22. This suggests that SNOT-22, compared to CRS-PRO, may encompass a wide range of symptoms that might not be relevant to all CRS patients. As a result, some items may not be disease-specific or reflect the most important aspect of a patient’s disease progression or treatment result.

This study presents a comprehensive analysis of CRS biomarkers using factor analysis to identify and quantify underlying inflammatory patterns and their association with disease severity and long-term outcomes. A key strength is the utilization of nasal secretions rather than tissue, permitting the capture of a wide range of inflammatory mediators longitudinally. Factor analysis reduced biomarker complexity, revealing four distinct factors related to Type 1, Type 2, and Type 3 inflammation, as well as a novel macrophage component. The longitudinal design, with assessments at multiple time points before and after ESS, allows for the evaluation of biomarker changes and their predictive value for long-term clinical outcomes.

We recognize that this study has limitations. This study was conducted at a single institution, although five different surgeons ascertained outcomes, and patients were invited to return after ESS for study visits independent of clinical need. Furthermore, this study identifies associations; it does not establish causal relationships. The longitudinal associations, while significant, are relatively moderate, indicating the influence of other unmeasured factors on long-term CRS outcomes. Additionally, we acknowledge that, although our longitudinal design incorporated three time points, future studies utilizing additional and more discrete postoperative intervals could provide greater resolution into the temporal evolution of inflammatory changes. Furthermore, patients receiving perioperative systemic steroids were not excluded from this study. While this reflects real-world clinical practice, it may introduce variability in inflammatory profiles. This should be considered when interpreting comparisons with other endotyping studies that may exclude such patients. Finally, while we present significant associations between factor scores and CRS outcomes, these are largely unadjusted and do not fully account for potential interactive effects, independent associations, or measured confounding that could be explored in future multivariate analyses. Nonetheless, the application of factor analysis to CRS biomarkers is a key step in understanding the underlying inflammatory process of CRS and providing quantitative measures that may aid with disease prognosis and treatment specificity.

## 5 | Conclusion

Factor analysis identifies distinct, quantifiable patterns of inflammation in CRS, offering improved associations with cross-sectional and longitudinal outcomes compared to individual biomarkers. This study revealed four factors, Type 1, Type 2, Type 3, and macrophage-associated, each exhibiting unique associations with CRS phenotypes and outcomes. T2 and M factors were elevated in CRSwNP patients and decreased post-surgery, highlighting the therapeutic impact of ESS. Future studies should explore the predictive value of these factors in treatment response and their role in guiding personalized CRS therapies.

## Supplementary Material

Supplementary Figure 3 **Supplementary Figure 3: Longitudinal correlations between V0 factors and V1 radiographic and patient-reported measures of disease severity**. The intensity of red and blue strength increase as strength of correlation becomes positive or negative, respectively, as represented in the heatmap. Spearman R values are shown in the center of the heatmap, and significance is shown in the bottom right corner. *p < 0.05; **p < 0.01; ***p < 0.001, ****p < 0.0001. N=148.

Supplementary Figure 2 **Supplementary Figure 2: Correlation between change in factor score and change in clinical outcome measures**. The intensity of red and blue strength increase as strength of correlation becomes positive or negative, respectively, as represented in the heatmap.

Supplementary Figure 1 **Supplementary Figure 1: Scree plot demonstrating optimal number of factors**: It shows the eigenvalues on the y-axis and the number of factors on the x-axis. The “elbow” or inflection point in the scree plot identifies 4 optimal factors. FA = Factor analysis.

Additional supporting information can be found online in the Supporting Information section.

## Figures and Tables

**TABLE 1 |:** Demographics, comorbidities, and V0 outcome comparison among control, CRSsNP patients, and CRSwNP patients.

	Control	CRSsNP	CRSwNP	Total
*n*	52	88	63	203
Demographics				p
Age, mean (SD)	46.37 (15.5)	45.9 (15.2)	46.3 (14.0)	1.00
Sex, *n* (%)				
Female	27 (51.9)	48 (54.5)	33 (52.4)	
Male	24 (46.1)	40 (45.4)	30 (47.6)	
Transgender (M to F)	1 (1.9)	0 (0.0)	0 (0.0)	
Race, n (%)				**0.01**
Asian	2 (3.8)	7 (7.9)	6 (9.5)	
Black or African American	7 (13.5)	**5 (5.7)**	**10 (15.9)**	
Decline to answer or missing	8 (15.4)	5 (5.7)	1 (1.6)	
White or Caucasian	35 (67.3)	71 (80.7)	46 (73.0)	
Ethnicity (%)				0.07
Declined to answer or missing	7 (13.5)	3 (3.4)	4 (6.3)	
Hispanic or Latino	5 (9.6)	4 (4.5)	8 (12.7)	
Not Hispanic or Latino	40 (76.9)	81 (92.0)	51 (81.0)	
Comorbidities				
Asthma [prior/current, *n* (%)]	10 (19.2)	40 (45.5)	37 (58.7)	**0.002**
AERD [yes, *n* (%)]	0 (0.0)	0 (0.0)	7 (11.1)	**<0.001**
Smoking [prior/current, n]	12 (23.1)	21 (23.9)	15 (23.8)	1.00
Allergic rhinitis, *n* (%)	16 (30.8)	43 (48.9)	31 (49.2)	**0.04**
Revision ESS, *n* (%)	NA	4 (4.5)	2 (3.2)	0.69
V0 outcomes				
LM total [mean (SD)]	NA	9.8 (4.1)	15.8 (4.0)	<**0.001**
SNOT-22 total [mean (SD)]	NA	46.4 (19.9)	47.3 (16.1)	0.79
CRS-PRO total [mean (SD)]	NA	25.9 (8.6)	26.0 (8.9)	0.96

*Note:* Data were analyzed using a one-way ANOVA for three groups, independent *t*-tests for comparisons between two groups, and chi-square tests for categorical variables. Statistical significance was set at *p* < 0.05.

Abbreviations: AERD, aspirin-exacerbated respiratory disease; CRS-PRO, Chronic Rhinosinusitis Patient-Reported Outcomes measure; CRSsNP, chronic rhinosinusitis without nasal polyps; CRSwNP, chronic rhinosinusitis with nasal polyps; ESS, endoscopic sinus surgery; LM, Lund–Mackay score; NA, not applicable; SNOT-22, 22-item Sinonasal Outcomes Test; V0, pre-surgery baseline/visit 0.

**TABLE 2 |:** Median and range for factor scores among healthy controls, CRSsNP patients, and CRSwNP patients at V0 and V1.

	Control	CRSsNP	CRSwNP	
>nu	>52	>88	>63	>*p*-value
>Pre-ESS (V0) (*N* = 203)	>Median (range)	>Median (range)	>Median (range)	
T1	−0.253 (−1.683 to 1.492)	0.015 (−1.017 to 1.494)	−0.120 (−1.049 to 1.651)	**0.008**
T2	−0.451 (−1.160 to 1.558)	−0.207 (−1.166 to 1.611)	0.613 (−1.186 to 2.701)	<**0.001**
T3	−0.253 (−1.683 to 1.492)	−0.121 (−1.596 to 2.505)	0.300 (−1.420 to 2.535)	**0.002**
M	−0.310 (−1.824 to 2.600)	−0.115 (−1.959 to 2.346)	0.421 (−1.673 to 2.738)	<**0.001**
6–12 months post-ESS (V1) (*N* = 149)		Median (range)	Median (range)	
*N*		86	63	*p*-value
T1	NA	−0.123 (−0.802 to 1.216)	0.0187 (−0.813 to 1.279)	0.94
T2	NA	−0.346 (−1.136 to 1.711)	−0.057 (−1.139 to 3.102)	0.06
T3	NA	−0.178 (−1.550 to 1.438)	0.071 (−1.686 to 1.937)	0.21
M	NA	−0.275 (−1.858 to 2.422)	−0.017 (−1.831 to 2.373)	0.09

*Note:* Kruskal–Wallis tests were used to compare medians between three groups, and Mann–Whitney *U* tests were used to compare medians between two groups. Statistical significance was set at *p* < 0.05.

Abbreviations: CRSsNP, chronic rhinosinusitis without nasal polyps; CRSwNP, chronic rhinosinusitis with nasal polyps; ESS, endoscopic sinus surgery; M, macrophage-associated; NA, not applicable; T1, Type 1; T2, Type 2; T3, Type 3; V0, pre-surgery baseline/visit 0CRSsNP, chronic rhinosinusitis without nasal polyps; CRSwNP, chronic rhinosinusitis with nasal polyps; ESS, endoscopic sinus surgery; M, macrophage-associated; NA, not applicable; T1, Type 1; T2, Type 2; T3, Type 3; V0, pre-surgery baseline/visit 0.
