# Supplementary figures and images for: Factor Analysis‐Based Quantitative Endotyping Improves Associations With CRS Cross‐Sectional and Longitudinal Outcomes
Source: Int Forum Allergy Rhinol. 2025 Sep 26;16(1):32–42. doi: 10.1002/alr.70027 (PMC12755909; doi:10.1002/alr.70027)

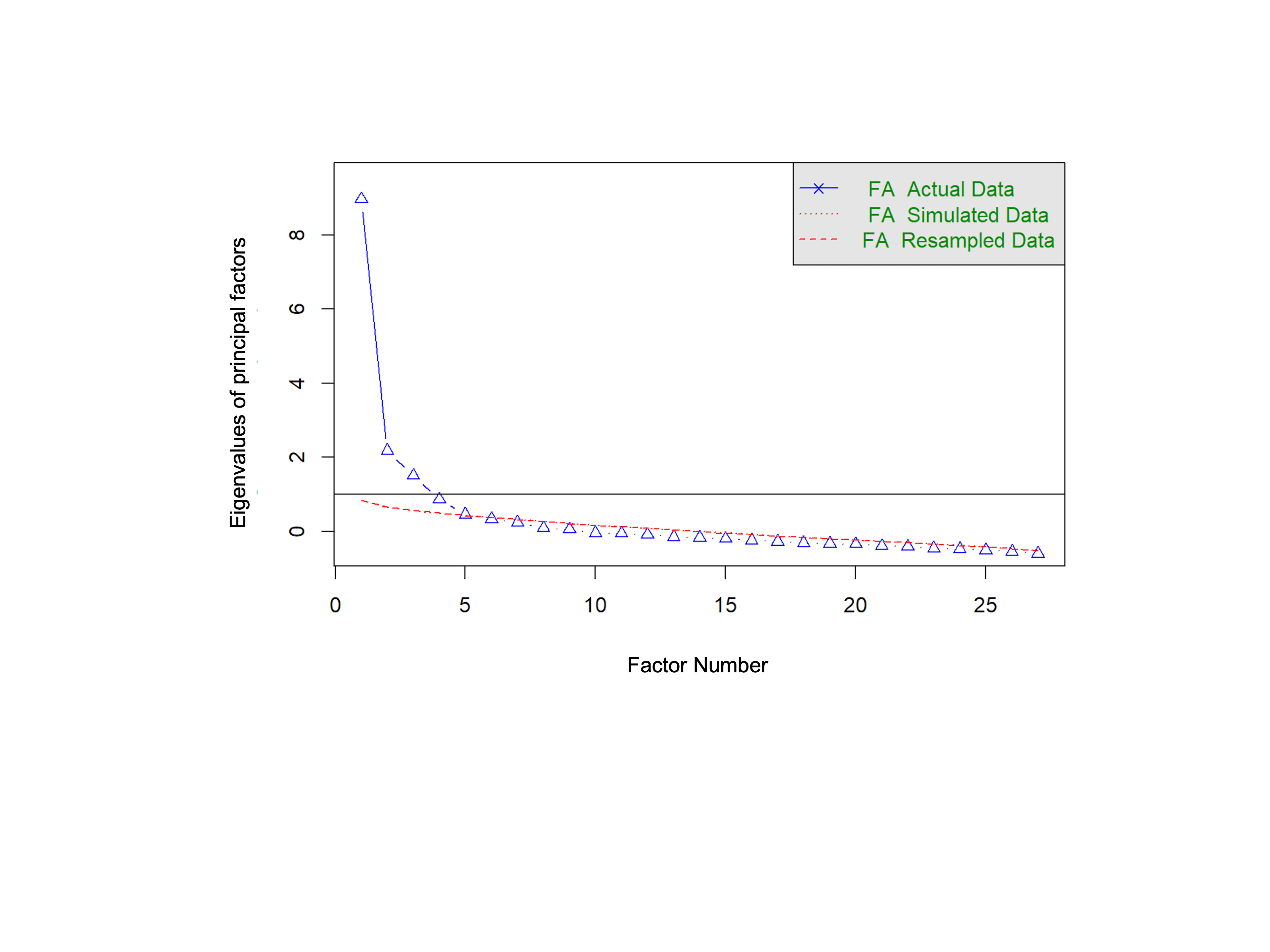

Supplement: Supplementary file 1 — Supplementary Figure 1: Scree plot demonstrating optimal number of factors: It shows the eigenvalues on the y‐axis and the number of factors on the x‐axis. The “elbow” or inflection point in the scree plot identifies 4 optimal factors. FA = Factor analysis. [file ALR-16-32-s003.tiff]

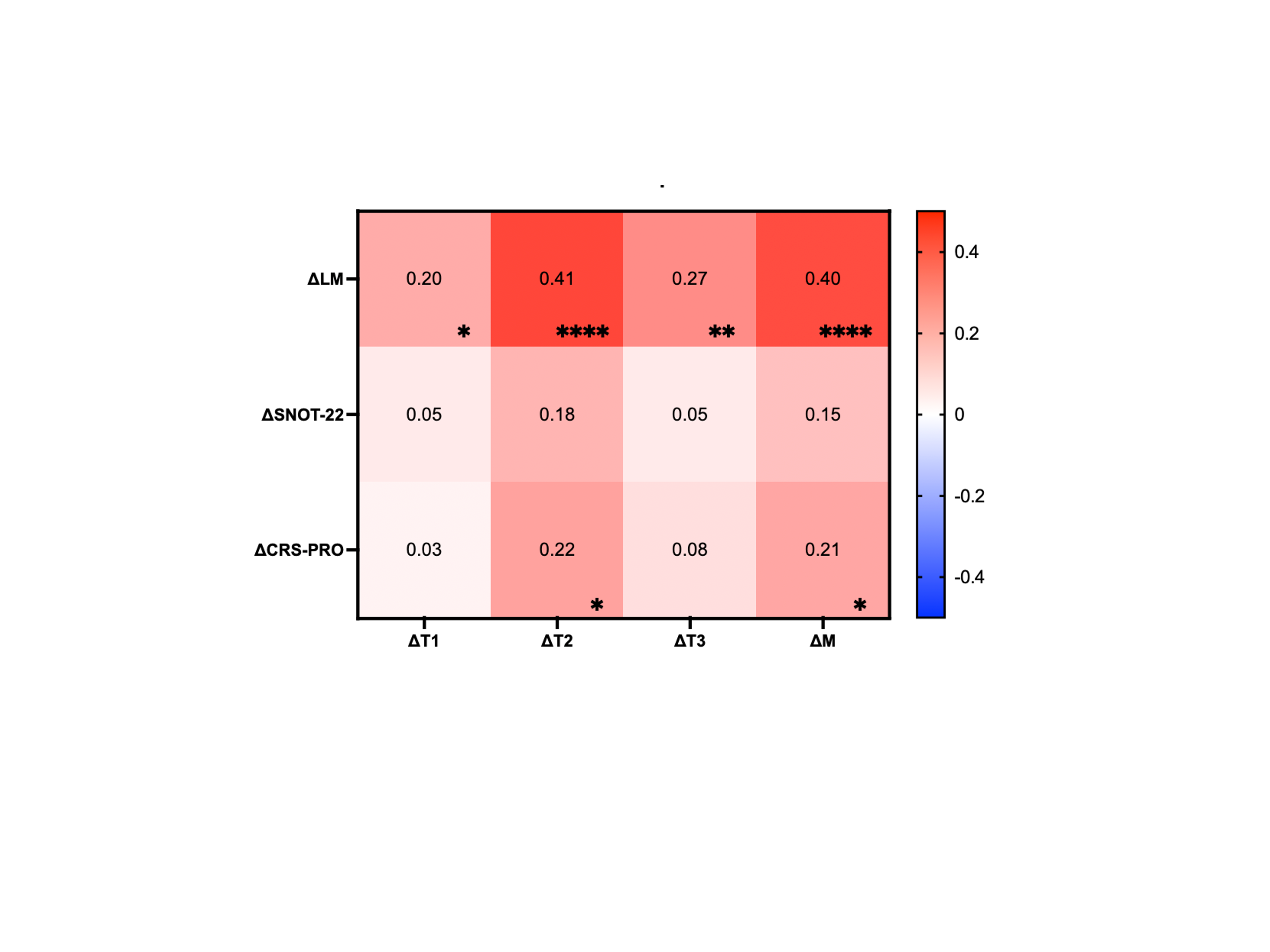

Supplement: Supplementary file 2 — Supplementary Figure 2: Correlation between change in factor score and change in clinical outcome measures. The intensity of red and blue strength increase as strength of correlation becomes positive or negative, respectively, as represented in the heatmap. [file ALR-16-32-s001.tiff]

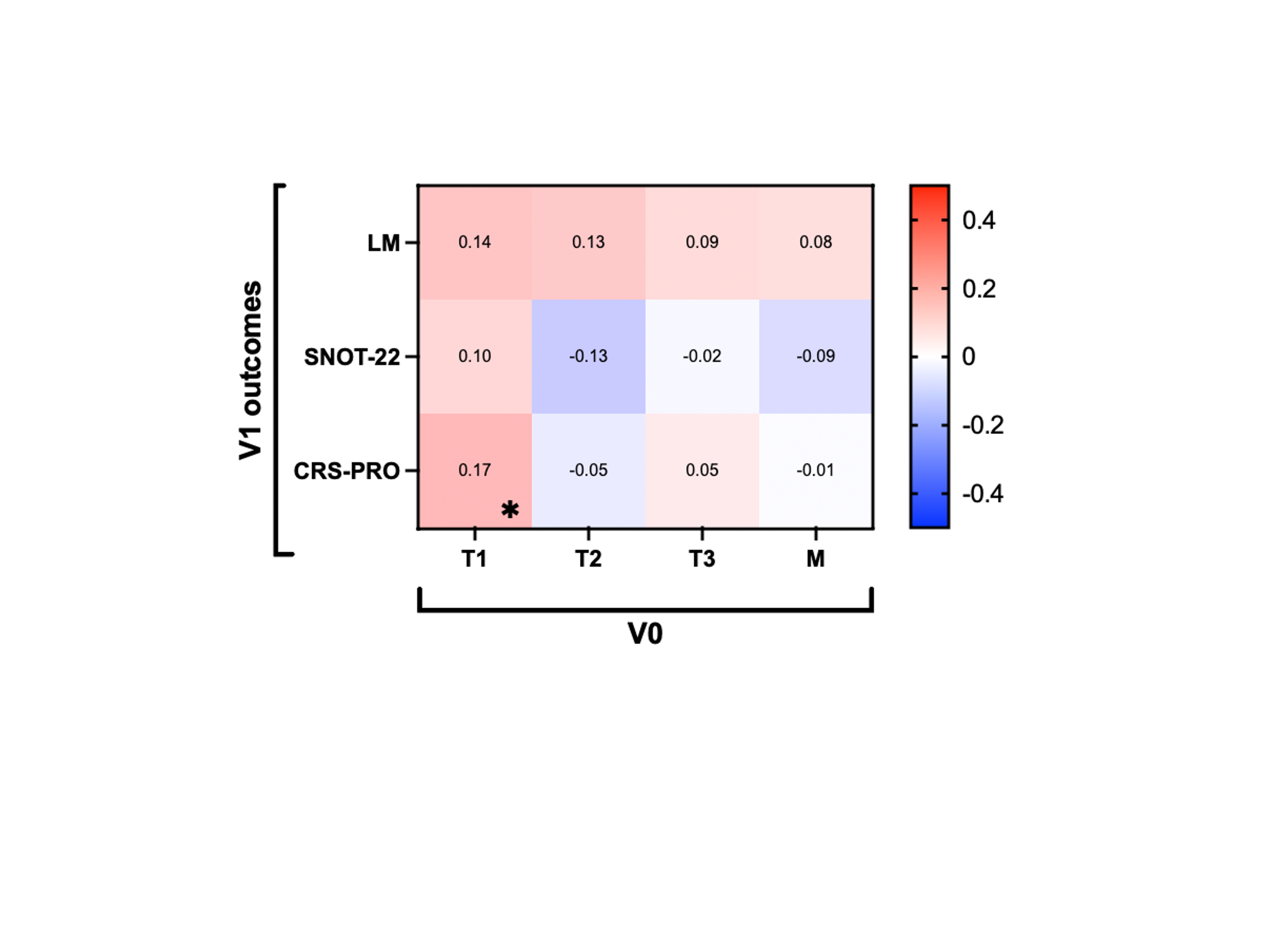

Supplement: Supplementary file 3 — Supplementary Figure 3: Longitudinal correlations between V0 factors and V1 radiographic and patient‐reported measures of disease severity. The intensity of red and blue strength increase as strength of correlation becomes positive or negative, respectively, as represented in the heatmap. Spearman R values are shown in the center of the heatmap, and significance is shown in the bottom right corner. *p < 0.05; **p < 0.01; ***p < 0.001, ****p < 0.0001. N=148. [file ALR-16-32-s002.tiff]
